# School Bullying and Post-traumatic Stress Disorder Symptoms: The Role of Parental Bonding

**DOI:** 10.3389/fpubh.2019.00075

**Published:** 2019-04-09

**Authors:** Stefanos Stylianos Plexousakis, Elias Kourkoutas, Theodoros Giovazolias, Kalliopi Chatira, Dimitrios Nikolopoulos

**Affiliations:** Department of Psychology, University of Crete, Rethymno, Greece

**Keywords:** bullying, victimization, parental bonding, post-traumatic stress disorder symptoms, trauma

## Abstract

Much research on school bullying and victimization have outlined several individual, family, and school parameters that function as risk factors for developing further psychosocial and psychopathological problems. Bullying and victimization are interrelated with symptoms of psychological trauma, as well as emotional/ behavioural reactions, which can destabilize psychosocial and scholastic pathways for children and adolescents. The current study explored the various dimensions of psychological trauma (depressive symptoms, somatization, dissociation, avoidance behaviours) associated with school bullying/victimization in relation to parental bonding among 433 students (8–16 years old) from representative large cities in Greece. The following scales were employed: (a) Olweus Bully/Victim Questionnaire, (b) Child Report of Post-traumatic Symptoms (CROPS), and (c) Parental Bonding Inventory instrument (PBI). Pathways analysis extracted a series of models which showed that maternal and paternal overprotection (anxious-controlling/aggressive) had positive association with post-traumatic stress symptoms. Specifically, the quality of parental bonding was related with children's bullying/victimization experiences and post-traumatic symptomology. Conversely, results indicated that maternal and paternal care can reduce the manifestation of post-traumatic stress symptoms. Implications for interventions are discussed.

## Introduction

Bullying is a significant social stressor for children and adolescents and has an estimated prevalence depending on the definition of bullying and the sample used. Peer maltreatment is estimated to be between 20 and 45%; bullying that occurs once a week or more can be as high as 32% (1–7). Bullying is defined as a long-lasting and systemic form of interpersonal aggression from an individual (perpetrator), where the victims are persistently exposed to negative or violent actions from other student/s over a period and struggles to defend themselves against these actions (8).

Bullying behaviours emerge as a consequence of repeated exposure to aggression in the form of verbal hostility, teasing, physical violence, or social exclusion (7). As a subtype of aggressive behaviour, bullying involves an imbalance of power between perpetrators and victims, where one side (perpetrator) demonstrates negative actions, and the other (victim) is not able to defend her-/himself (9–12). Many researchers have emphasized the group aspect of bullying not only as a dyadic problem between a bully and a victim which is rather recognized as a group phenomenon including bystanders (13, 14).

Exposure to bullying is a significant risk factor that contributes independently to the emergence of psychological difficulties and pathology, regardless of pre-existing mental health symptomology, genetic predisposition, or family psychosocial difficulties (15). Many studies have found that bullying is the root of severe negative psychological and physical consequences, including depression, anxiety, reduced self-esteem, decreased school attendance and avoidance symptoms, somatization, as well as suicide ideation/attempts/completions (3, 4, 10, 11, 16–18). Some researchers claim that school bullying can cause symptoms such as those experienced by survivors of child maltreatment and abuse (19). Bullying prevalence in Greece has the same varying statistics as schools in other European countries. Owing to increasing prevalence of bullying incidents in Greece, with consequent emotional difficulties to many students, there is an emerging necessity to carry out research exploring the deleterious effects (including trauma symptoms) both to victims and bullies (20). In fact, this is the first proposed study in Greece, with a randomly selected sample, to examine post-traumatic stress symptomology resulting from bullying and victimization in relation to parental bonding.

Much research has revealed that various individual, family, and school parameters function as risk factors for developing further psychosocial and psychopathological difficulties. Symptoms of psychological trauma, as well as emotional/ behavioural reactions, can destabilize children's psychosocial and scholastic pathways. Previous studies have emphasised the catalytic role of dysfunctional families, and more specifically the negative impact of hostile and aggressive parental overprotection, on children's involvement in bullying incidents, both as victims or perpetrators. On the other hand, maternal and/or paternal care is also a significant protective factor against the manifestation of children's internalizing or externalizing disorders (21, 22). Thus, the present study examined whether bullying experiences, as a victim or perpetrator, is associated with post-traumatic stress symptoms (PTSD, DSM-V). More importantly, we addressed whether parental bonding quality (care, indifference, overprotection, encouragement of autonomy) influences the relationship between bullying roles and traumatic symptoms.

### Bullying and Negative Psychological Effects

Extensive research has revealed that bullying experiences are associated with emotional difficulties, including feelings of loneliness, anxiety, depression, adjustment difficulties, low academic performance, low self-esteem (4, 23–33) and a lack of appropriate social skills. More recent studies have shown that bullying is also associated with symptoms of psychological dissociation (34–36), somatization (37–39), avoidance behaviours (40–46), and symptoms of PTSD (47–50).

### Bullying and Trauma

Although the initial introduction of PTSD in the DSM was not primarily designed with children and adolescents in mind, a developmental perspective has gradually been introduced in newer DSM versions (51). The evolution of diagnostic criteria has indicated that PTSD in childhood and adolescence is almost identical with criteria applied to adults (i.e., trauma re-enactment, horrible dreams, “shrinked” hope for the future, avoidance of stimuli associated with the trauma event, lack of interest, and somatization of stress and anxiety; (52). Bullying can potentially hamper bio-psycho-social growth; therefore, it is necessary to apply a developmental perspective to increase our understanding as to how a PTSD diagnosis can be applied, as well as examine how we can reduce emerging traumatic effects (53). Bullying is an interpersonal event that occurs at a salient relational level; from this perspective, there are several aspects of children's and adolescents' lives that could be affected in a deleterious way as a result of PTSD experiences. Such experiences occur at a very critical time, when the brain is developing bio-psycho-social systems that regulate emotions, dramatically influencing behaviour (54). In recent years, some studies have examined the degree to which school bullying is related to the presence of PTSD symptomology (55). For instance, Mynard et al. (30) revealed that 37% of bullying victims reported significant PTSD symptoms. Additionally, River (56) revealed that 25% of participants appeared to experience PTSD symptoms, particularly intrusive memories of bullying instances, even after leaving school. McKenny et al. (57) also found a significant association between being bullied and PTSD symptoms among school-aged children. Furthermore, several studies observed that bullies, who may also be victims themselves, experience higher levels of suicidal ideation and potential PTSD symptomology (4, 5, 58).

Herman [1992, (59)], as well as Terr (60), claimed that an individual who has been repeatedly bullied, experiences a situation of helplessness, similar to a victim of trauma; therefore, they suggested that bullying dynamics could be considered a form of repeated trauma. Olweus (8) emphasized that three distinct criteria characterize bullying into an experience of chronic trauma. Primarily, there is an intention to harm the victim, which is directly experienced by a victim or indirectly by a bystander. Bullying can be direct or indirect and cause both physical and psychological damage. Secondly, the repetitive nature of bullying is similar to Terr's (60) trauma of a second type, as well as Herman's complex PTSD (59). The accumulative effect of exposure to bullying undermines a victim's sense of self and can cause short-term and long-term consequences. Finally, bullying involves an imbalance of power between the perpetrator and victim, which can lead a victim into a sense of helplessness and weakness, critical characteristics in all forms of trauma (61). Several studies provide evidence of a strong association between bullying and PTSD symptoms, emphasizing that bullying could potentially be a form of continuous trauma rather than a mere acute stress experience (62, 63).

### Bullying and Parental Bonding

Previous studies have emphasized the impact of dysfunctional family relationships with both parents, which are linked with a child's involvement with bullying behaviours (64, 88). It is now clear that both bullies and victims experience inadequate support from parents. There are also significant findings indicating the existence of domestic violence and other adversities within the lives of bullies and victims (65). Many bullies appear to experience authoritarian parental styles and conflicts (66). Authoritarian style parental bonding is strongly associated with higher levels of bullying, while passive style parental bonding and pedagogy are linked with victimization (67). More recent findings indicate that among parents who practice hostile control, children exhibit a higher potential for engaging in bullying behaviours (22, 68). Children who experience insecure and avoidant parental bonding are likely to demonstrate callous-unemotional characteristics (69, 70). Parents who lack care and emotional warmness can rear children who exhibit a lack of empathy, which can lead to bullying proneness (20). Recent studies have also shown that bullying perpetrators experience low levels of parental care and higher levels of overprotection (71). Parents who are supportive and demonstrate a caring style reduce the possibility of their children engaging in bullying behaviours (72). Other studies have emphasized the significance of a father's protective role as a defence against peer bullying (21). A father's involvement is an even more important factor when a mother's involvement is low (21).

## The Present Study

Until recently, very few studies have investigated the relationship between bullying and traumatic symptoms, while accounting for the role of parental bonding. This is quite striking given that many children and adolescents are frequently exposed to bullying behaviours that result in serious emotional symptoms that can destabilize their academic, emotional, and social progress. Therefore, we reviewed previous literature in order to investigate aspects of post-traumatic symptoms related to bullying behaviours among school-aged children. There has been a call for more studies clarifying the effect of bullying on the manifestation of post-traumatic stress symptoms and how parental bonding affects this relationship (30, 55–57) within a representative sample of Greek students. Based on previous studies (51), we expected that as many as 20–30% of students would report a bullying experience, but fewer would report frequent bullying. Although previous studies have revealed that more boys than girls are involved in bullying, this appears to have changed as different indirect forms of bullying are emerging (2, 41). The core question of our study was to clarify the degree to which bullying behaviours are associated with symptoms of PTSD. Regarding gender, previous studies have revealed that girls are more vulnerable to manifesting post-traumatic stress symptoms (73, 74); therefore, we expected to find higher post-traumatic symptom scores among girls than boys (4, 5).

We explored the various aspects/dimensions of psychological trauma symptoms in relation to school bullying/victimization along with parental bonding quality (care, indifference, overprotection, and encouragement of autonomy) among 433 students (8–17 years old) from all over Greece. Specifically, we examined how traumatic symptom levels (depression, somatization, avoidance behaviour, dissociation) are associated with parental bonding type, in the context of bullying type, and how bullying behaviour roles are shaped (75). Here, we discussed and analysed a specific model that emphasises how certain types of parental bonding can cause certain emotional reactions in relation to bullying and traumatic symptoms. One basic assumption is that there is a negative role of parental overprotection (anxious or controlling/aggressive) and emotional reactions/risk for victimization that emerges for students in the present sample.

## Method

### Sample and Procedure

We used a randomly selected sample taken from a survey conducted in Greece during 2013–2014. We selected a sample from schools from the largest urban areas of Greece, including Athens (65.6%, *n* = 284), Thessaloniki (21.0%, *n* = 91), and Crete (13.4%, *n* = 58). A total of 433 students aged 8–17 years participated in our study. Sample size was estimated apriori using G^*^Power version 3.1 (76). The analysis indicates that a sample size of 311 would be sufficient to detect significant direct and indirect associations with a power of 0.80 and an alpha of 0.05 (The total population was 516.034 students).

Boys comprised 45.5% (*n* = 197) of our sample, while girls comprised 54.5% (*n* = 236). The age distribution of our sample was as follows: 10 years (8.1%, *n* = 35), 11 years (18.9%, *n* = 82), 12years (21.0%, *n* = 91), 13 years (20.1%, *n* = 87), 14 years (20.6%, *n* = 89), 15 years (10.6%, *n* = 46), 16 years (0.5%, *n* = 2), and 17 years (0.2%, *n* = 1). The class distribution was as follows: Primary School: 5th Grade (24%, *n* = 104), 6th Grade (20.1%, *n* = 87); High school: 1st Class (22.4%, *n* = 97), 2nd Class (21.7%, *n* = 94), 3rd Class (11.8%, *n* = 51).

Consent to carry out the study was initially obtained from the Ministry of Education (both for primary and secondary education departments). Later, we sought permission from local school authorities and finally from each school's directors. Written informed consent was sought from parents to allow their children to participate. We sent a sealed letter to each parent (through their children) with a written description regarding the nature and goals of our study and invited parents to provide written consent. Following guidelines from the British Association for Counselling and Psychotherapy Code of Ethics (77), children who did not obtain written permission from parents were excluded from participating. We asked the class teacher to ensure children that all responses would be confidential, so that children could feel secure and confident about participating. We clearly told the children that no individual from the school or their parents would see their answers; this was to ensure each student's psychological integrity and obtain answers provided with a sense of safety and security. Our survey met all ethical standards and criteria of the Greek Educational Department from the Ministry of Education, as well as the Ethical Research Committee at the University of Crete. Due to the sensitive nature of our study concerning trauma symptoms and bullying, we used methods that respected children and ensured their health and safety. We stated clearly the goals of our study, we only included children who obtained permission from their parents and the school director, the language and research instruments were child-friendly, and we stated clearly that no child was obliged to participate without his/her individual permission (77).

### Instruments

Bullying was measured using the Olweus Bully/Victim Questionnaire (78, 79). The students were first provided a written definition/explanation of bullying behaviours so as to assist with comprehending the phenomenon. The scale comprised 40 questions, which sought information regarding the following areas: Prevalence of bullying, duration of the bullying event, type of bullying, identification of bullying roles, general psychosocial adjustment, internalizing, or externalizing symptoms, self-evaluation, depressive tendencies, general aggressiveness, antisocial behaviours, general bullying attitudes, and evaluation of awareness regarding teachers and parents. (7, 78, 79). Our sample completed the Greek version of the Revised Olweus Bully/Victim Questionnaire (78, 80, 81). Cronbach alphas ranged between 0.83 and 0.87 for victimization across groups and time measures and between 0.65 and 0.88 for bullying (81).

Symptoms of trauma were measured using the Children's Report of Post-traumatic Symptoms (CROPS) (109). CROPS is a self-report scale comprising 25 questions examining a child's post-traumatic stress symptoms from having been involved in a stressful incident. Answers were provided on an analogue scale ranging from 0 to 2 (three-point Likert scale, where 0 = never, 1 = sometimes, and 2 = very often). A total score was estimated by adding all answers together, where higher scores meant the existence of clinically significant PTSD symptomology (82). The following main traumatic symptoms were examined: 1. depression/anxiety (Questions: 4, 7, 9, 10, 11, 16, 19, 20, 21, 22, 23, 25) 2. psychic-dissociation (Questions: 1,2,3,17,18) 3. somatisation (Questions: 12, 13, 14,15), 4. avoidance behaviours (Questions: 5, 6, 8, 24). As for CROPS we also used the Greek Version (67). The CROPS has internal consistency with an alpha value of 0.91. Its 4–6 week test–retest correlation is 0.80 (83, 84).

Parental bonding was measured using the Parental Bonding Inventory Scale (PBI) (75, 85). The scale consists of two questionnaires: (a) Father's Parental Bonding and (b) Mother's Parental Bonding. Both questionnaires comprise 25 questions, and each explore the following factors: 1. care (Questions: 1, 5, 6, 11, 12, 17), 2. over-protection (Questions: 8, 9, 10, 13, 19, 20, 23), 3. indifference (Questions: 2, 4, 14, 16, 18, 24), 4. encouragement of autonomy (Questions: 3, 7, 15, 21, 22, 25).

### Statistical Analyses

Data were analysed using the SPSS/V21 statistical program (86, 87) for Windows. Statistical methods included frequency and percentage analyses, means comparisons, hypothesis testing, parametrical tests, *t*-tests for independent samples, ANOVAs, regression analyses, path analyses, and confirmatory factor analyses.

**Figure 1:**
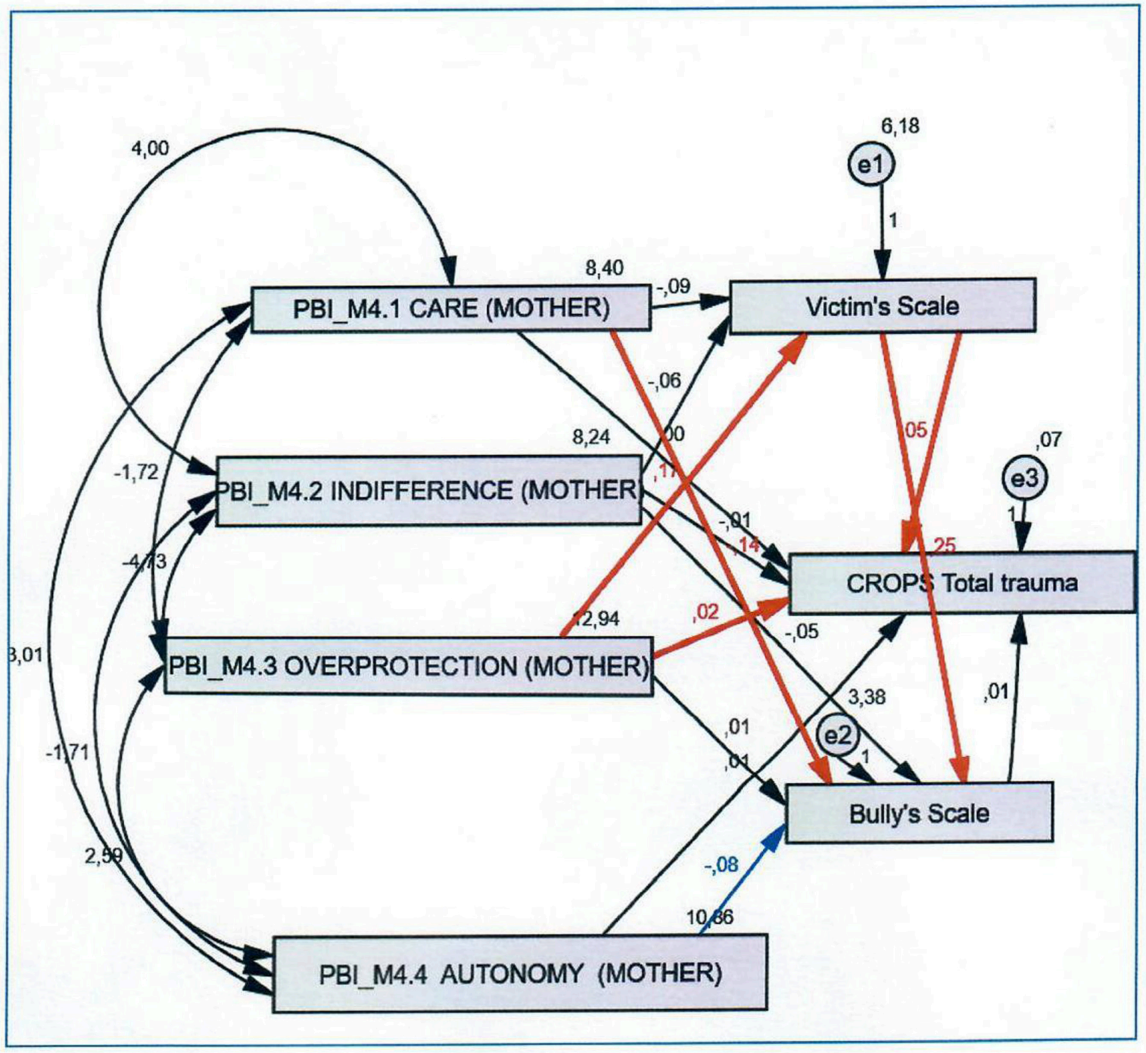
CROPS total trauma and mother's behavior.

**Figure 2:**
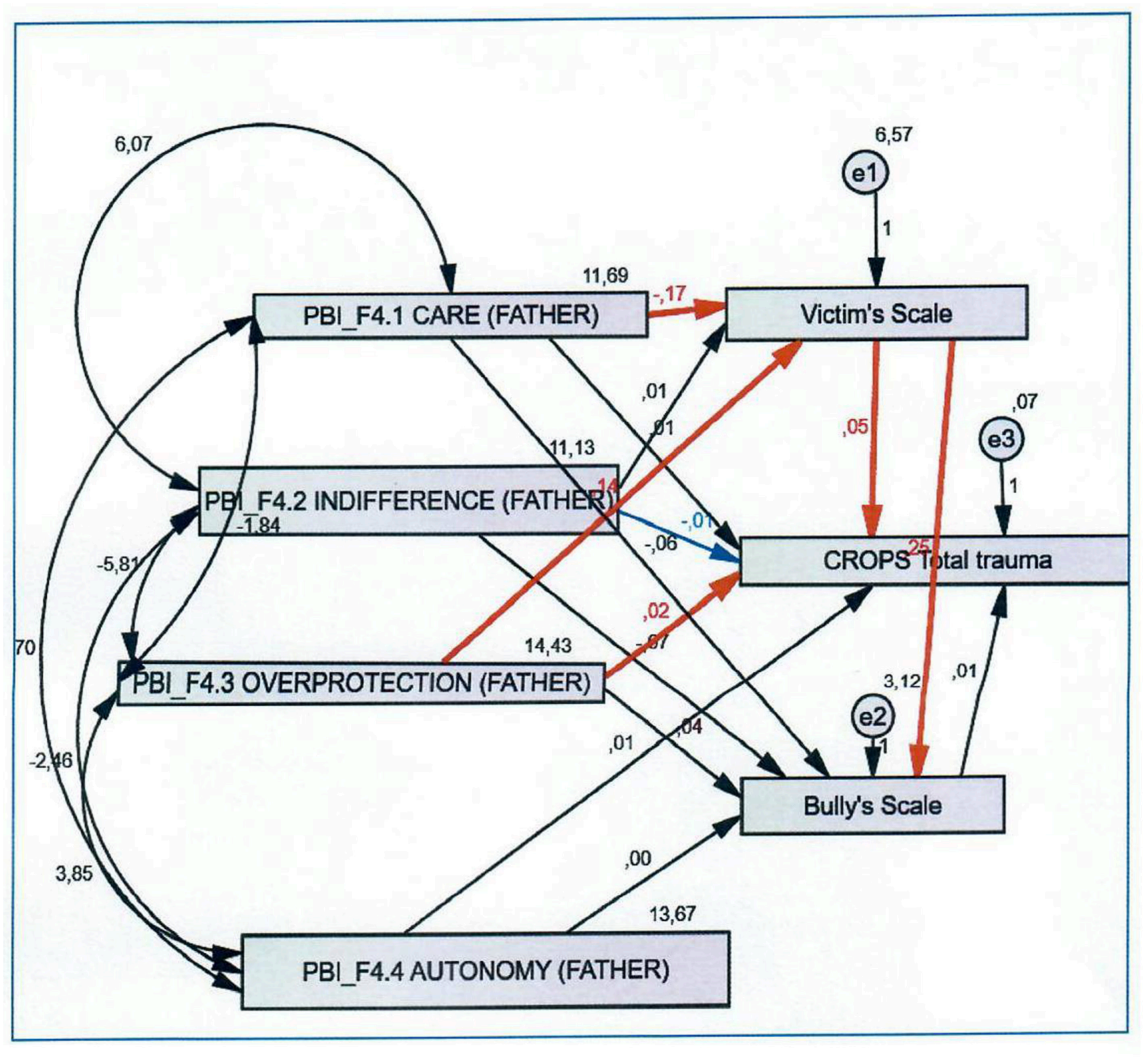
CROPS total trauma and father's behavior.

**Figure 3:**
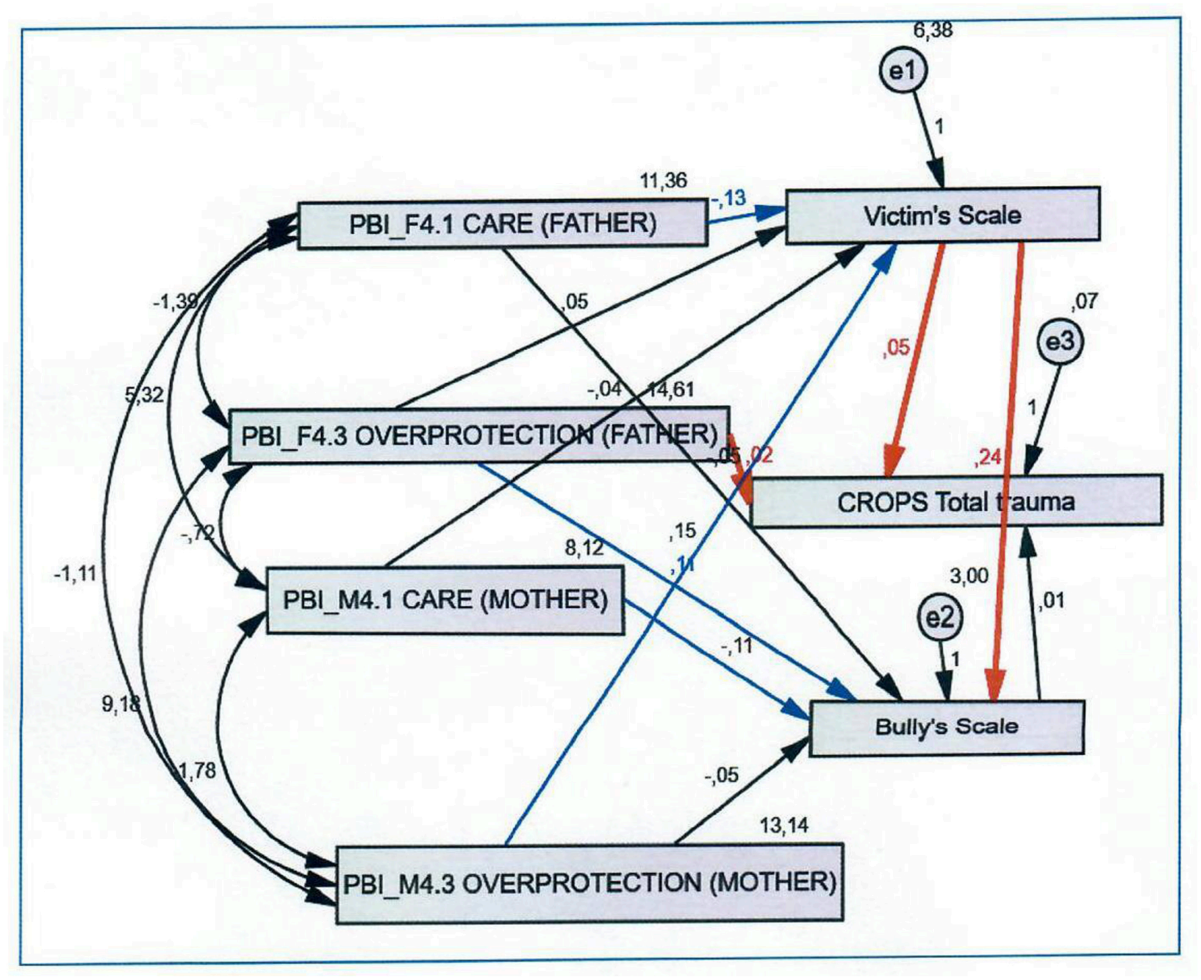
CROPS total trauma, father's and mother's care, and overprotection.

Confirmatory analyses revealed that the identified factors fit the data well. Path analyses generated a series of models (see Figures 1–3) with the following parameters: (a) four dimensions of parental bonding (care, indifference, overprotection, encouragement of autonomy) (b) four types of traumatic symptoms (depression-anxiety, dissociation, avoidance, somatization), and (c) two forms of bullying (bully, victim).

Our data fit all necessary conditions for best-fit models. The aforementioned results agree with the assumptions of a confirmatory factor analysis. We can assume that the PBI and CROPS factors in our data are consistent with those mentioned in previous studies and similar theories. The thresholds listed in the tables are taken from Hu and Bentler (88).

## Results

Our results revealed that 102 students (23.5%) experienced bullying (as victims) at least once during the last year. A total of 14 students (3.2%) reported high frequency bullying (one to several times per week) as seen in Table 1. A total of 6 students (1.4%) reported that they had experienced physical bullying, or one of the more prevalent forms of bullying, many times every week, while 38 students (8.8%) reported that they had experienced bullying only once or twice every week. As expected, verbal bullying, as well as relational bullying, was the most prevalent form of bullying. More specifically, 27 students (6.3%) reported that they had experienced verbal bullying one or more times every week, while 52 students (12%) had experienced relational bullying. Racial bullying was reported by 24 students (5.5%). Several students (*n* = 68, 15.7%) reported being bullied by classmates (Table 2). Regarding gender, victims reported that perpetrators were mainly boys (45 students, 10.4%), but there was a significant number of girl perpetrators reported (n = 12, 2.8%) (Table 3). Our findings are consistent with previous studies demonstrating that perpetrators are, to a large degree, boys. Bullying occurred mainly at the individual level rather than at the group level. At the group level, 31 students (7.2%) reported being bullied by boys, while 15 students (3.5%) reported being bullied by girls. However, 18 students (4.2%) reported being bullied by both boys and girls. It is very important to mention that a significant number of students (*n* = 26, 6%) did not react (e.g., telling perpetrators to stop or asking for help from peers or an adult); therefore, these victims manifested intense emotional reactions (e.g., crying). A significant number of students were not able to report bullying behaviours to an adult in order to protect themselves; specifically, 28 students (6.5%) did not tell anyone.

**Table 1:** Students from survey's sample who were bullying victims by type of bullying and frequency of the event.

**Type of Bullying**	**Bullying frequency**					
	**1 or 2 times per year**		**2 or 3 times per month**		**Once or more times per week**	
	**Frequency**	**Percent (%)**	**Frequency**	**Percent (%)**	**Frequency**	**Percent (%)**
Physical	76	17.6	10	2.3%	16	3.7
Racial	24	5.6	4	0.9	5	1.2
Verbal	90	20.8	34	7.9	27	6.3
Relational	97	22.5	19	4.4	22	5.1
All Types	142	32.9	44	10.2	55	12.7

**Table 2:** Students from survey's sample who reported being bullied by classmates or other students.

**Perpetrators class**	**Bullying Victims**	
	**Frequency**	**Percent (%)**
Classmates	68	15.9
Higher class	17	4.0
Lower Class	2	0.5
Different classes	13	3.0

**Table 3:** Students from survey's sample who reported being bullied by number of perpetrators.

**Number of perpetrators**	**Bullying victims**	
	**Frequency**	**Percent (%)**
Mainly 1 boy	45	10.5
Mainly 1 girl	12	2.8
Some boys	31	7.2
Some girls	15	3.5
Both boys and girls	18	4.2

It is important to highlight that several students (*n* = 73, 16.9%) reported that teachers never spoke to them about bullying in school (Table 4). Additionally, 41 students (9.5%) reported that parents did not speak to them about bullying. Regarding where at school bullying occurred, 50 students (11.6%) reported being bullied while coming to, or leaving from, school. It is quite interesting to note students' perceptions about teachers' awareness. For instance, 78 students (18.0%) stated that teachers were not aware of bullying behaviours, and 35 students (8.1%) felt that teachers did not try to stop the bullying. Regarding the role of bystanders, many students (*n* = 98, 22.6%) reported that their peers very rarely intervened, even though most students (96.1%) feel that bullying is a very distressing experience. Regarding bystanders' reactions, while many students (*n* = 172, 39.7%) reported the incident to an adult, it is quite interesting that several students (*n* = 142, 32.8 %) tried to have no relationship with the event, while some students participated or watched with pleasure (*n* = 17, 3.9%).

**Table 4:** Students from survey's sample by way of reaction and feelings for bullying and their view for others behavior.

**Students way of reaction and feelings for bulling and their view for others behavior**	**Students from sample**	
	**Frequency**	**Percent**
Didn't tell to anyone after bulling event	28	6.5
Feel that bulling is a very distressing experience	416	96.1
Teacher's never spoke about bulling	73	16.9
Teacher's not aware about of bulling behaviors	78	18.0
Teacher's did not try to stop the bulling	78	18.0
Parents never spoke about bulling	41	9.5
Peers very rarely intervened in bulling	98	22.6

Our *t*-test analyses revealed that the average values for boys as victims were higher than girl's for different types of bullying (physical, verbal, relational, racial). For the general victim's scale, the difference between boys and girls was statistically significant (with boys having a higher average).

Using a psychometric instrument (CROPS) for the identification of post-traumatic stress symptoms, we observed that 100 students (23.1%) (Table 5) reported that they “daydream during the day”, indicating the existence of emotional difficulties related to internalized problems of anxiety, depression, and psychic dissociation, which are the most serious manifestations of trauma reactions (83). Additionally, 155 students (35.8%) reported that sometimes “I lose track of myself when people talk to me”. Several students (144; 33.3%) reported serious difficulties with their ability to concentrate, which is indicative of internalized difficulties due to anxiety or depression. Additionally, 112 students (25.9%) reported feeling sad and melancholic.

**Table 5:** CROPS results by variable (Frequencies & Percentages).

**Crops variables**	**Never**		**Sometimes**		**Too many times**		**Total**	
	**Frequency**	**%**	**Frequency**	**Percent**	**Frequency**	**%**	**Frequency**	**%**
CROPS__1. I daydream	147	34.2	183	42.6	100	23.3	430	100.0
CROPS__2. I “space out” when people are talking to me	244	56.7	155	36.0	31	7.2	430	100.0
CROPS__3. I find it hard to concentrate	251	58.6	144	33.6	33	7.7	428	100.0
CROPS__4. I think about bad things that have happened	175	40.9	200	46.7	53	12.4	428	100.0
CROPS__5. I try to forget the bad things that have happened	117	27.5	173	40.6	136	31.9	426	100.0
CROPS__6. I avoid reminders of bad things that have happened	126	29.2	177	41.0	129	29.9	432	100.0
CROPS__7. I worry that bad things will happen	206	47.9	165	38.4	59	13.7	430	100.0
CROPS__8.I do special things to make sure nothing bad happens	159	37.4	135	31.8	131	30.8	425	100.0
CROPS__9. It is hard for me to go to sleep at night	298	69.5	92	21.4	39	9.1	429	100.0
CROPS_10. I have bad dreams or nightmares	262	60.6	141	32.6	29	6.7	432	100.0
CROPS_11. I do some things that I am probably too old for	254	60.0	132	31.2	37	8.7	423	100.0
CROPS_12.I get headaches	239	55.3	165	38.2	28	6.5	432	100.0
CROPS_13. I get stomachaches	298	69.1	112	26.0	21	4.9	431	100.0
CROPS_14. I feel sick or have pains	326	75.6	88	20.4	17	3.9	431	100.0
CROPS_15. I feel tired or low energy	198	46.3	185	43.2	45	10.5	428	100.0
CROPS_16. I feel all alone	337	78.6	72	16.8	20	4.7	429	100.0
CROPS_17. I feel strange or different than other kids	314	73.9	92	21.6	19	4.5	425	100.0
CROPS_18. I feel like there is something wrong with me	311	72.3	101	23.5	18	4.2	430	100.0
CROPS_19. I feel like it is my fault when bad things happen	233	53.9	171	39.6	28	6.5	432	100.0
CROPS_20. I am a jinx or bad-luck charm	336	78.0	78	18.1	17	3.9	431	100.0
CROPS_21. I feel sad or depressed	305	70.6	112	25.9	15	3.5	432	100.0
CROPS_22. I do not feel like doing much	285	66.3	125	29.1	20	4.7	430	100.0
CROPS_23. Things make me upset or mad	125	29.0	241	55.9	65	15.1	431	100.0
CROPS_24. I am on the lookout for bad things that might happen	170	39.6	182	42.4	77	17.9	429	100.0
CROPS_25. I am nervous or jumpy	222	51.9	166	38.8	40	9.3	428	100.0

It is noteworthy that 200 students (46.2%) reported that “sometimes, I think about the awful things that have happened to me,” indicating the existence of traumatic stress by reliving the traumatic event. Another important finding was that 173 students (40%) reported that “sometimes I try to forget about the awful things that have happened to me,” reflecting the traumatic symptom of avoidance. A total of 165 students (38.1%) reported that “I am concerned that awful things could happen to me,” indicating a struggle with anxiety. Anxiety was also identified by 92 students (21.2%) who reported sleep difficulties. Another 141 students (32.6%) reported sleep disturbances with nightmares. It is notable that 165 students (38.1%) reported the existence of headaches; 112 students (25.9 %) reported having stomach aches; and 88 students (20.3%) reported sometimes feeling sick. These three somatic symptoms are part of the axis of somatization for PTSD (52). A total of 185 students (42.7%) reported feeling tired and lacking in energy, and 72 students (16.6%) reported feeling completely alone, further indicative of depression symptomology. Additionally, 92 students (21.2%) reported feeling “strange and differently than other children,” and 101 students (23.3%) reported feeling that “something is wrong with me,” reflective of possible anxiety or depression symptoms. A total of 171 students (39.5%) reported feeling that “it is my fault when awful things happen to me,” while 78 students (18.0%) reported “feeling bad-luck” in their life. Additionally, 125 students (28.9%) felt that they were not of great importance. These responses are indicative of possible low self-esteem and a generally bleak future, which are all characteristic of PTSD. It is also interesting that 241 students (55.7%) reported that several things triggered annoyance and anger, possibly reflecting externalizing difficulties with anger management. Finally, 182 students (42%) reported being vigilant to awful things that could occur, suggesting another key post-traumatic stress symptom (52).

While average symptom scores were higher among girls when compared with boys, only avoidance behaviour scores were statistically significant (*p* < 0.05).

### Path Analysis for Mothers' Behaviours in Relation to Total Trauma

Figure 1 revealed that having an overprotective mother was positively associated with the emergence of traumatic symptoms (*r.f*. = 0.02, *p* < 0.001); however, an ideal level of maternal care reduced the likelihood of a child being a bully (*r.f*. = −0,14, *p* < 0.001). Having also an overprotective mother was related with a child being a victim (*r.f*. = 0.17, *p* < 0.001). Additionally, being a child victim was strongly associated with the emergence of traumatic symptoms (*r.f*. = 0.05, *p* < 0.001) and with the possibility of being a bully (*r.f*. = 0.25, *p* < 0.001). We also observed that a mother's facilitation of autonomy reduced the likelihood of a child being a bully (*r.f*. = −0,08, *p* < 0.05). In terms of bullying roles and depression, the following were observed. First, a child who experienced an overprotective mother was more likely to be victim (*r.f*. = 0.017, *p* < 0.001). Several of these children exhibited symptoms of depression (indirect effect) (*r.f*. = 0.05, *p* < 0.001), while another segment also reported being a bully (*r.f*. = 0.025, *p* < 0.001). These results suggest that a mother's overprotective tendencies and traumatic experiences as a victim are plausible risk factors for the exhibition of depressive symptoms (internalizing symptoms) and/or aggressive bullying (externalizing symptoms). Furthermore, results suggested that a lack of maternal care was increasing the likelihood of a child being a victim (*r.f*. = −0.014, *p* < 0.001).

Bullying roles in relation to psychic dissociation produced the following observations. There was a significant positive association between mother's overprotection and child victimization (indirect effect) (*r.f*. = 0.017, *p* < 0.001). Greater maternal overprotection increased the likelihood of a child being a victim. Being a child victim was also strongly related with the possibility of being a perpetrator (indirect effect) (*r.f*. = 0.025, *p* < 0.001). A lack of maternal care was also related with a child taking on a perpetrator role (*r.f*. = −0.014, *p* < 0.001), with a caring mother making it less likely that a child would be a bully. Being a victim was also linked to the manifestation of psychological dissociation (*r.f*. = 0.05, *p* < 0.001). Also, a lack of maternal encouragement of a child's autonomy was associated with a child being a perpetrator (*r.f*. = −0.08, *p* < 0.05), with the converse being true if a mother encouraged autonomy. Maternal overprotection was also related with a child experiencing psychological dissociation.

Bullying roles in relation to somatization produced the following results. First, there was a significant positive association between maternal overprotection and child victimization, and being a victim was strongly related with being a perpetrator (indirect effect) (*r.f*. = 0.025, *p* < 0.001). Conversely, maternal care reduced likelihood of a child being a perpetrator (*r.f*. = −0.014, *p* < 0.001), establishing maternal care as a major protective factor. Being a victim had positive association with reported somatization (indirect effect) (*r.f*. = 0.06, *p* < 0.001). Maternal encouragement of a child's autonomy was also related to a decreased likelihood of a child being a perpetrator (*r.f*. = −0,08, *P* < 0,05), while maternal overprotection was significantly associated with a child's somatization (*r.f*. = 0.02, *p* < 0.05).

Assessing bullying roles in relation to avoidance behaviour led to the following observations. Maternal overprotection had positive association with child victimization (indirect effect) (*r.f*. = 0.017, *p* < 0.001), and being a victim had strong relation with a child being a perpetrator (*r.f*. = 0.025, *p* < 0.001). Increased maternal care was related to a decreased likelihood of a child being a perpetrator (*r.f*. = −0.014, *p* < 0.001). Being a victim had also positive association with avoidance behaviours (*r.f*. = 0.04, *p* < 0.001). Furthermore, maternal overprotection was strongly related with a child manifesting avoidance behaviour (*r.f*. = 0.03, *p* < 0.001). Maternal facilitation of a child's autonomy was associated with a decreased likelihood of a child being a perpetrator (*r.f*. = −0.08, *p* < 0.05). Finally, maternal care was related to a decrease in a child's avoidance behaviours.

Within the somatization and avoidance behaviour path models, maternal overprotection was associated of children taking on a victim role. Furthermore, maternal overprotection directly related with the manifestation of children's traumatic symptoms. This was particularly the case for avoidance behaviours.

### Path Analysis for Fathers' Behaviours in Relation to Total Trauma

Figure 2 indicates that having an overprotective father is strongly associated a child being a victim (*r.f*. = 0.14, *p* < 0.001). Conversely, paternal care had negative relation with being a victim (*r.f*. = −0.17, *p* < 0.001). Being a child victim was also positively associated with a child being a bully (*r.f*. = 0.25, *p* < 0.001). Furthermore, being a child victim had strong relation with reported trauma symptoms (*r.f*. = 0.05, *p* < 0.001). Having an overprotective father was additionally related with reported trauma symptoms (*r.f*. = 0.02, *p* < 0.001). A father's indifference had negative association with reported trauma symptoms (*r.f*. = −0.01, *p* < 0.05). For the path analysis regarding bullying roles and depression, the following was observed. First, a lack of paternal care was related with a high likelihood of a child being a victim (*r.f*. = −0.17, *p* < 0.001), the child reporting depression symptoms (*r.f*. = 0.05, *p* < 0.001), and the child being a perpetrator (*r.f*. = 0.014, *p* < 0.001). As compared to the maternal path analysis, a paternal lack of care had positive association with a child being a perpetrator. Second, paternal overprotection increased the probability of a child being a victim (*r.f*. = 0.05, *p* < 0.001) and subsequently experiencing depression symptoms (*r.f*. = 0.025, *p* < 0.001).

In terms of bullying roles and psychological dissociation, the following were observed. Specifically, paternal overprotection was positively associated with a child being a victim (*r.f*. = 0.014, *p* < 0.001). Being a victim had strong relation with a child being a perpetrator (*r.f*. = 0.025, *p* < 0.001). On the contrary, paternal care had negative association with being a child victim (*r.f*. = −0.017, *p* < 0.001). Furthermore, being a victim was positively related with reported psychological dissociation (indirect effect) (*r.f*. = 0.06, *p* < 0.001).

Regarding the path between bullying roles and somatization, we first observed a significant positive association between paternal overprotection and child victimization (*r.f*. = 0.014, *p* < 0.001). Child victimization was also strongly related with perpetration (*r.f*. = 0.025, *p* < 0.001). Paternal care was a protective factor against victimization (*r.f*. = −0.017, *p* < 0.001). Victimization, in turn, was related with reported somatization (indirect effect) (*r.f*. = 0.06, *p* < 0.001). Hence, paternal overprotection increases the probability that a child would become a victim and later manifest somatization and/or perpetrator behaviours. This same pathway was observed when examining a father's lack of care.

Regarding the path between bullying roles and avoidance behaviours, we observed a significant positive association between paternal overprotection and victimization (indirect effect) (*r.f*. = 0.014, *p* < 0.001). Child victimization was related with perpetration (*r.f*. = 0.025, *p* < 0.001). Conversely, paternal care was negatively associated with victimization (*r.f*. = −0.017, *p* < 0.001). Victimization was positively related with reported avoidance behaviours (*r.f*. = 0.04, *p* < 0.001). Finally, paternal overprotection was associated with avoidance behaviours (*r.f*. = 0.02, *p* < 0.001). Hence, victimization was related with avoidance behaviours through paternal overprotection, while an indirect relationship between victimization and avoidance was still significant.

As shown in Figure 3, having an overprotective father is strongly related with the report of traumatic symptoms (*r.f*. = 0.02, *p* < 0.001). Second, a child who has been a victim is more likely to report traumatic symptoms (indirect effect) (*r.f*. = 0.05, *p* < 0.001). Third, being a child victim increased the probability of being a bully (*r.f*. = 0.24, *p* < 0.001). Table 3 revealed that paternal care decreased the likelihood of a child being a victim (*r.f*. = −0.13, *p* < 0.05). Maternal overprotection had a significant association with victimization (*r.f*. = 0.15, *p* < 0.05) Paternal overprotection was related with a child expressing bullying behaviours (*r.f*. = 0.11, *p* < 0.05), while, maternal care decreased this probability (*r.f*. = −0.11, *p* < 0.05).

## Discussion

The most significant finding regarding mothers' role, as perceived by the child, is that mother's overprotection (see Figure 1) has strong association with a child becoming a victim, with two different pathways/outcomes; first pathway is related with the development of traumatic symptoms and the second pathway with child to become a perpetrator. We can assume that mother's overprotection consists a significant risk factor for children's vulnerabilization that may lead to traumatic symptoms or for a child to react in an aggressive way through bullying behavioral patterns (15). A plausible explanation of maternal overprotection negative association is that this type of parenting practice (in the hostile/controlling or anxious form) seen to impede children to spontaneously developing their own potential (22).

Our second significant finding is that a lack of maternal care, which indicates blunted emotional responsiveness, was related with a child becoming a perpetrator. Similarly, previous studies, showed that lack of care, which considered a form of emotional deprivation and neglect, is associated with instrumental or intentional aggressive behaviour (89, 90). Studies have revealed that parent's emotional absence, accompanied with lack of emotional responsiveness, creates a state of intense emotional frustration that can transform into negative and hostile feelings such as anger and rage, resulting to hostile behaviours and open aggressiveness (21, 22).

Similarly, regarding father's role (see Figure 2), the strongest finding is that paternal overprotection is associated without any other contributing factor with the manifestation of traumatic symptoms. The role of father, as a key socialization factor, is critical during transition in pre-adolescent or adolescent period (21, 22). We assume that fathers who seem to impede their children's primordial need for socialization through overprotection (in the aggressive or anxious controlling form) have a very strong negative impact on their children's developmental pathway, with increased risk of leading them to trauma symptoms. Additionally, father overprotective attitude is related, to a significant degree, with the bully behaviour. A plausible explanation for this finding is that some children and adolescents react in an aggressive way against these paternal practices or transform their hostile and aggressive feelings to externalized symptoms/aggressive behaviours. Another significant finding regarding father's role is that the lack of paternal care which also means lack of paternal protection and support, increases the likelihood of these children for being victimized and in turn to either develop traumatic symptoms or aggressive behaviour (bully) [see also (15)].

It is worth noticing that according to our results (see Figure 3) a father's overprotective stance has a stronger association than mother's on a child's emotional state and vulnerabilization, as it is related directly to symptoms of trauma. According to previous studies (21, 22), a plausible explanation is that father has a more critical role than mother, during preadolescence and adolescence, on children's social/emotional development and coping strategies formation. Our results show that overprotective fathering reduces their children's psychological potential, rendering them vulnerable undertaking bullying roles and exhibiting traumatic symptoms.

The PBI though, does not differentiate controlling or aggressive from anxious overprotective parenting. However, research shows that both forms of overprotective parenting are considered a form of emotional abuse in the sense that prevent children from critical socialization process and therefore from developing the appropriate interpersonal skills and coping strategies (22, 91).

Overall, the most significant finding of our research was the negative association of parental overprotection, on children's involvement to bullying and victimization, as well as to the development of trauma states, through different pathways, a finding which is consistent with other studies (22). Both overprotection (as a form of control) and lack of care (as a form of emotional neglect and lack of support) regarding social-emotional development, create high risks conditions for psychological vulnerability. When the child experiences additional forms of victimization in other contexts, such as school, she/he is likely to develop strategies to cope with intrapersonal and interpersonal anxiety and negative emotions, subsequently resulting in internalizing or externalizing symptoms (aggressiveness or depression/emotional-social withdrawal). Our results are consistent with previous studies which have emphasized the impact of dysfunctional relationships with both parents, which are linked with a child's involvement with bullying behaviours [66; (88); 41]. Moreover, our results are consistent with recent studies who have also shown that perpetrators experience low levels of parental care and higher levels of overprotection (71). Our research findings are in agreement with those which emphasized the significance of a father's protective role as a defence against peer bullying (15, 21) and generally about caring parents who are supportive and demonstrate a caring style of parenting that reduces the possibility of their children engaging in bullying behaviours (72).

The quality of parental bonding plays an essential role in children's affective and psychosocial development and related disorders (22). In our study, one of the most significant findings regarding both parents overprotective attitude, as perceived by the children, is that consists a significant risk factor for being involved in bullying and victimization and in developing traumatic symptoms through various pathways.

Considering these results in totality, we believe that it is necessary to create a new integrative approach (92–94) of examining bullying through the lens of traumatic symptoms and the quality of parental bonding. This helps provide an assessment of deeper psychological interactions that lead to the emergence of negative emotional consequences among victims and bullies, therefore, to be able to design and establish a more comprehensible model of prevention and intervention within family and school contexts that will take into consideration the quality of family dynamics and the quality of parental bonding.

**Figure 4:**
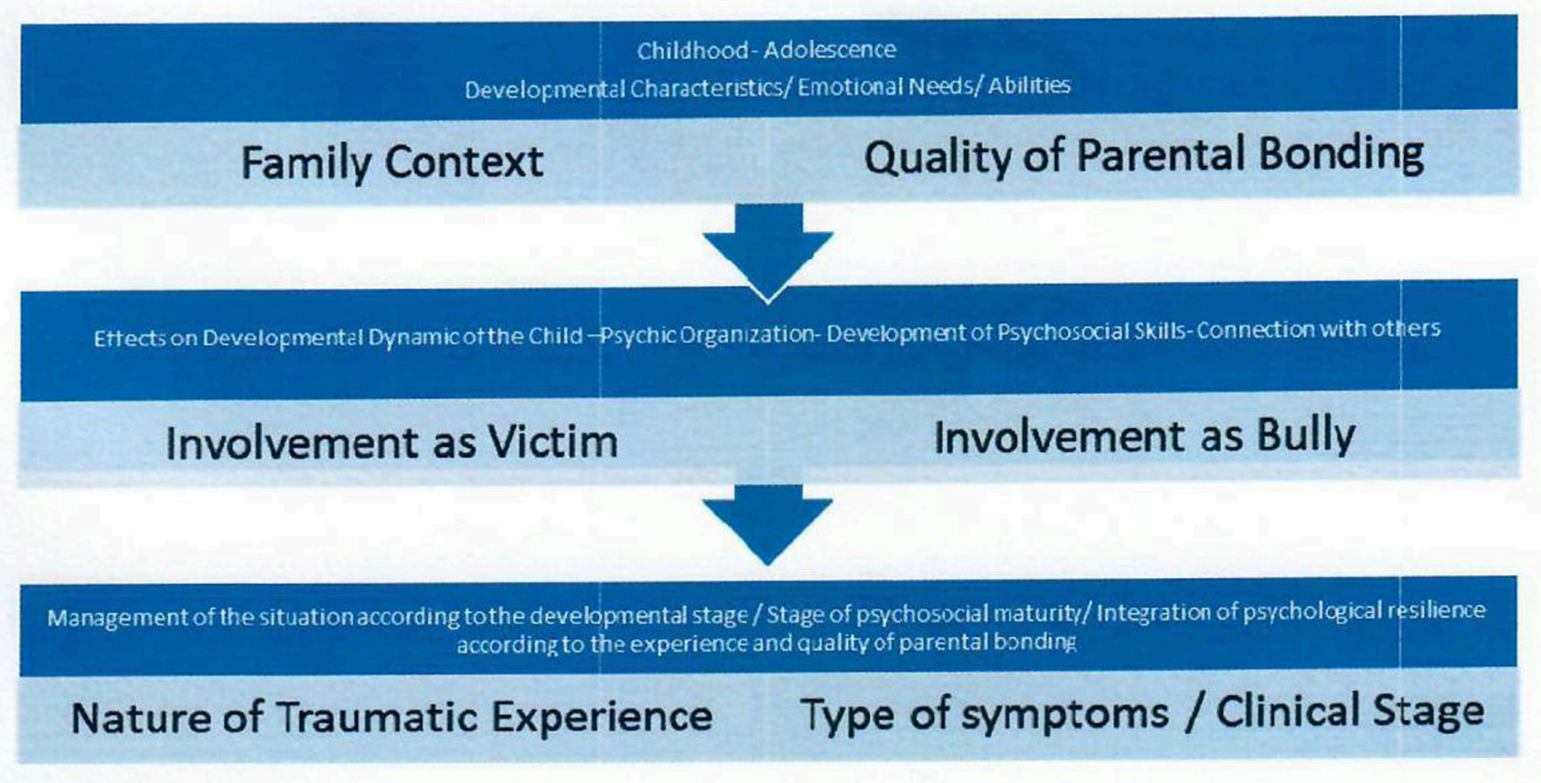
Bullying through the lens of traumatic relationships.

We also suggest that holistic approaches for tackling bullying should incorporate other experiential interventions (108, 113) (i.e., through stories, painting, music, art interventions), which could facilitate children to create a coherent narrative of their painful experiences, through indirect and alternative therapeutic methods, especially for those who have manifested traumatic symptoms in response to bullying and neglect. Therefore, attempts should be made to help children regain their self-esteem, a sense of emotional control and core identity by helping them better cope with family and school based interpersonal trauma. Hence, we argue that bullying can cause multiple traumatic symptoms, especially when is combined with problematic or dysfunctional family background that create a vulnerability to children or lead them to aggressive counteractions (95). Consequently, our therapeutic interventions should consider the bullying experience as a form of relational/interpersonal trauma (94) that should be placed in the context of previous family relational experiences that play an essential role as protective or risk factors (15). It is important to highlight the significant role of the therapeutic relationship when confronting trauma symptoms, so as to develop the appropriate therapeutic strategies according to the child's developmental stage (see Figure 4) or pathway and specific family context in order to re-establish trust and ensure post-traumatic growth.

We attempted to achieve a new understanding regarding bullying phenomena through the lens of traumatic relationships in order to emphasize that bullying comprises an interpersonal trauma that occurs between individuals or groups (63). One individual who has been bullied experiences a state of helplessness and weakness, similar to any other victim of a very traumatic experience, especially if these occurs in the context of very important relationships, such as parental and peer relationships, and during critical developmental stages, when children or preadolescents have not yet completed/integrated the appropriate cognitive-emotional mechanisms to properly deal with stressful situations like bullying. Therefore, it has been suggested that bullying dynamics are experienced as repeated trauma (59, 60, 112). Our study is consistent with previous research results indicating that stressful life events do play a crucial role in the development of depression (96, 97), anxiety (98), and post-traumatic stress symptoms (99). The present study also highlights the need to include problematic parental practices and bonding as stress factors that lead to victimization and bullying, often without any other factor mediation to post-traumatic stress symptoms.

Our study also emphasizes that even children who react in aggressive ways, as bullies, might have experienced problematic or destabilizing relationships with parents. A plausible clinical explanation, advanced by many experts and researchers is that aggressive and bully behaviour in this case consists a coping strategy/defence mechanism against feelings of vulnerability or even depression and low self-esteem (33). Unfortunately, bullying is often considered a normal developmental experience by several school directors and staff (62). However, the association between bullying and post-traumatic stress symptoms is considered a form of trauma (62). Several health professionals insist that children and adolescents who are exposed to extreme stress are more likely to develop serious mental health issues; therefore, bullying is very often a continuous trauma rather than an acute stressful experience (63).

We propose a therapeutic model for addressing bullying that includes post-traumatic stress symptoms; this is based on a previous model, namely the diathesis-stress model, that has received significant empirical evidence (100, 101) and has contributed to our understanding of how stressful events in the context of relationships can result in depression outcomes (102) and social exclusion (81). We do believe that bullying comprises an important stressful event that causes serious emotional disturbances to children and adolescents, regardless of the bullying role (bully, victim, bully/victim, bystander) and manner of involvement (26, 27). Our clinical intervention and hermeneutical model is also consistent with previous results, including Ferguson and Dyck's (103) and Dishion's (95) studies, who argued that it is critical to apply a model that explores the complex relational patterns within family and school contexts and considers the stress and emotional states of a child in order to better clarify the development of aggressiveness.

Bullying is not merely a dyadic problem between a bully and a victim but is rather recognized as a group phenomenon occurring in a social context where several factors operate to facilitate, prevent or hide bullying behaviours (13, 14). Our research is placed within the social/ecological model of school bullying focusing on the quality of parental bonding which is strongly related post-traumatic symptoms (104–107, 110).

As long as this is the first proposed study in Greece, to examine post-traumatic stress symptomology resulting from bullying and victimization in relation to parental bonding, we believe that our research results can have many useful implications for practice and improve bullying situation in Greek Schools, while percentages fluctuate in similar levels as other European countries.

### Implications for Practice

We propose several implications for clinical and school practice considering the fact that most school interventions focusing on alleviating bullying experiences are currently ignoring the existence of PTSD symptoms. It is important to highlight that schools need to develop interventions to deal with traumatic symptoms in an appropriate way. Schools must focus on specific students who have manifested symptoms of trauma and provide psychoeducation programs. For instance, school staff could develop better awareness so as to identify the existence of post-traumatic stress symptoms in order to refer students to relevant services (i.e., individual/group therapy and/or educational interventions). School personnel could be more vigilant and sensitive to different forms of avoidance behaviours (typically higher among girls) that possibly mask a child's trauma from a bullying experience.

Every school could develop holistic and systemic programs that provide counselling and psychotherapy, as well as individual interventions, that can focus on a child's relationships with his/her family, internalizing or externalizing difficulties, and consider bullying as a form of interpersonal trauma.

### Limitations and Future Research

One of limitation was the self-report nature of our chosen methodology. Future research should also include qualitative methods (interviews, etc.) that engage bullies and victims so as to clarify a deeper understanding of bullying and parental attitude or family relational dynamics through children's and adolescents' personal narrative/experience and a discourse analysis methodology. It also appears that in the Parental Bonding Inventory, latent variables may be perceived differently across different age groups. Thus, further research is needed in order to understand whether such differences are due to actual developmental changes in children's perceptions of the parent-child relationship or conceptual problems pertaining to children's ability to conceive the PBI's theoretical constructs. Another limitation was the small number of perpetrators sampled. Future research should recruit larger samples in order to offer a more complete picture of bullying phenomena. Given that the present study was carried out for only 1 year, we cannot treat this as a longitudinal analysis. Future longitudinal research could explore risks and protective factors, in addition to victims' and bullies' personality characteristics that are relevant to development during a longer study period. Additionally, future research should explore the bi-directionality/causality of bullying. This indicates that we should clarify a crucial question: whether the manifestation of post-traumatic stress symptoms is the result of a bullying experience or if children who experienced trauma in the past are more likely find themselves in bullying situations.

Future research should be more analytical and qualitative in order to examine comorbidities and other essential elements, including family risk and protective factors and the perceived role of masculinity in a society, as boys typically display higher percentages of all forms of bullying. We also need to examine the effect of cultural issues and ethics, social norms, and the role of each therapeutic approach in order to address bullying in schools and the community.

## Data Availability

All datasets generated for this study are included in the manuscript and/or the supplementary files.

## Ethics Statement

Written informed consent was obtained from parents of participating adolescents and children. All participants provided written consent or assent before completing the questionnaires. The study protocol was approved by the University of Crete Ethics Committee and the Ministry of Education in Greece. All parents of subjects gave written informed consent in accordance with the Declaration of Helsinki.

## Author Contributions

SP, KC, TG, and DN designed the study which is part of PS Ph.D. thesis. SP wrote the first draft of the manuscript, and developed and performed the statistical analysis in conjunction with EK and TG. EK reviewed and edited the manuscript and approved the final version of the manuscript.

### Conflict of Interest Statement

The authors declare that the research was conducted in the absence of any commercial or financial relationships that could be construed as a potential conflict of interest.
